# Engineering Bipolar Interfaces for Water Electrolysis Using Earth-Abundant Anodes

**DOI:** 10.1021/acsenergylett.3c02351

**Published:** 2023-11-30

**Authors:** Andrew W. Tricker, Jason K. Lee, Finn Babbe, Jason R. Shin, Adam Z. Weber, Xiong Peng

**Affiliations:** † Energy Storage and Distributed Resources Division, Lawrence Berkeley National Laboratory, Berkeley, California 94720, United States; ‡ Department of Chemical & Biomolecular Engineering, University of California Berkeley, Berkeley, California 94720, United States

## Abstract

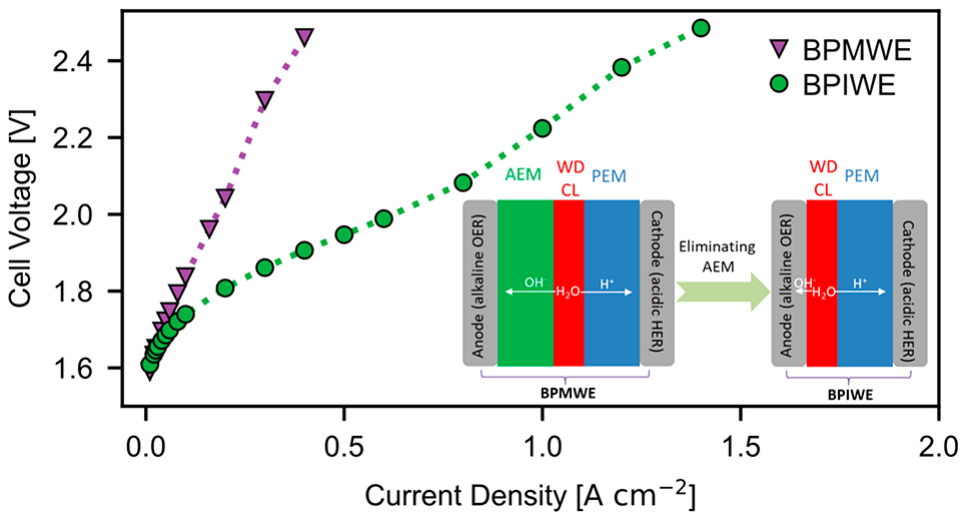

Developing efficient and low-cost water electrolyzers for clean hydrogen production to reduce the carbon footprint of traditional hard-to-decarbonize sectors is a grand challenge toward tackling climate change. Bipolar-based water electrolysis combines the benefits of kinetically more favorable half-reactions and relatively inexpensive cell components compared to incumbent technologies, yet it has been shown to have limited performance. Here, we develop and test a bipolar-interface water electrolyzer (BPIWE) by combining an alkaline anode porous transport electrode with an acidic catalyst-coated membrane. The role of TiO_2_ as a water dissociation (WD) catalyst is investigated at three representative loadings, which indicates the importance of balancing ionic conductivity and WD activity derived from the electric field for optimal TiO_2_ loading. The optimized BPIWE exhibits negligible performance degradation up to 500 h at 400 mA cm^–2^ fed with pure water using earth-abundant anode materials. Our experimental findings provide insights into designing bipolar-based electrochemical devices.

The growing demand for more sustainable approaches to utilizing energy and mitigating the global threats of climate change necessitates development of alternative energy carriers and industrial feedstocks, especially for sectors that are not easily adapted to direct decarbonization via intermittent renewable electrons.^1^ Electrolytic hydrogen, via water electrolysis, offers a promising medium to store renewable energy for long durations and can be used to decarbonize hard-to-decarbonize sectors such as steel/cement production,^2^ heavy-duty transportation,^3^ heavy-oil/biomass upgrading,^4,5^ ammonia synthesis,^6,7^ and industrial chemicals production.^8^ To accommodate the projected skyrocketing demand for hydrogen from 90 million tonnes per annum (Mtpa) in 2020 to over 500 Mtpa by 2050,^9^ low-cost and efficient water electrolyzers need to be developed and deployed at scale.

Commercial electrolyzers, such as proton-exchange-membrane water electrolyzers (PEMWEs) and liquid-alkaline water electrolyzers (LAWEs), are operated under either strongly acidic or alkaline conditions to minimize series resistance. However, the oxygen-evolution reaction (OER) and hydrogen-evolution reaction (HER), as the primary half-reactions on the anode and cathode, respectively, are not necessarily under optimal conditions due to non-coincident preference of electrolyte pH. Previous research has concluded that the anode kinetics of acidic OER (2H_2_O → 4H^+^ + O_2_ + 4e^–^, *E*^0^ = 1.23 V vs SHE) is more sluggish than that of alkaline OER (4OH^–^ → O_2_ + 2H_2_O + 4e^–^, *E*^0^ = 0.401 V vs SHE), due to the O–O bond formation being the rate-determining step in extreme acidic conditions.^10^ On the other side, alkaline HER (2H_2_O + 2e^–^ → 2OH^–^ + H_2_, *E*^0^ = −0.828 V vs SHE) is kinetically less favorable than acidic HER (2H^+^ + 2e^–^ → H_2_, *E*^0^ = 0 V vs SHE), due to the Volmer step becoming rate-limiting.^11,12^ For instance, the exchange current density measured on platinum catalyst is at least 2 orders of magnitude lower for alkaline compared to acidic HER.^13^ Besides affecting the kinetics, the operating pH has an additional influence on the electrode and cell design. On the PEMWE anode, an acidic electrolyte coupled with oxidative potentials results in a highly corrosive environment that mandates the use of platinum-group metals (PGMs) as catalysts and coatings for the bipolar plates and porous-transport layers (PTLs). Consequently, this leads to an anode-driven cost profile with extremely high capital expenditures for commercial PEMWEs.^14^ More critically, gigawatt-to-terawatt-scale deployment of PEMWEs is likely to be restricted by the global supply of iridium,^15^ which is the only practical anode catalyst for acidic OER. On the cathode, water consumption by alkaline HER limits the desired dry-cathode operation at high partial differential pressure (∼30 bar), as direct water feed to the cathode is necessary at industrial operating conditions. Therefore, it can be expected that an alkaline condition is desired for the anode, as it leverages a more robust alkaline OER and less corrosive reacting environment, which allows for the use of low-cost and earth-abundant materials, while an acidic condition is preferred for the cathode to produce high-purity and pressurized hydrogen by the kinetically more favorable acidic HER.

To this end, bipolar-membrane water electrolyzers (BPMWEs) have been proposed as an alternative to conventional monopolar ion-exchange-membrane water electrolyzers. Typical BPMWEs are operated in reverse-bias mode and are composed of an alkaline anode and acidic cathode, separated by a bipolar membrane (BPM), which is created by lamination of a proton-exchange membrane (PEM) and an anion-exchange membrane (AEM) with water dissociation (WD: H_2_O ↔ OH^–^ + H^+^) catalysts in between them. The performance and durability of BPMWEs have increased drastically in recent years, especially for those using the conventional “H-type” cell with supporting electrolyte.^16−18^ Although these results are promising, further improvements to the performance and longevity of BPMWEs are faced with challenges due to the ordinarily sluggish WD reaction. Both the electric field, illustrated by the second Wien effect, and the catalytic effect, contributed by a WD catalyst within the bipolar junction, are generally accepted to enhance the rate of WD.^17,19^ Whether BPMWEs have true advantages over incumbent PEMWEs and LAWEs or emerging technologies such as anion-exchange-membrane water electrolyzers relies on high-performing and durable demonstrations using earth-abundant materials under pure water (deionized water: 18.2 MΩ)-fed membrane-electrode assembly (MEA) configurations.

Here, we demonstrate an engineered bipolar-interface water electrolyzer (BPIWE) to achieve excellent water-electrolysis performance and durability using earth-abundant anode materials. It was found that a bipolar interface could be maintained even after elimination of the AEM in the conventional BPM configuration. We then explored the impact of loading TiO_2_ as a WD catalyst on BPIWE performance as well as the longevity of BPIWEs under intermediate currents. Our work provides a new pathway to develop efficient water electrolyzers using earth-abundant materials and insights into approaches to engineering bipolar interfaces for electrochemical devices.

The BPIWE consists of an alkaline anode, a PEM, and an acidic cathode, with a WD catalyst between the anode and PEM (Figure 1). At the anode and PEM interfaces, the WD reaction produces hydroxides (OH^–^) and protons (H^+^), which migrate to the anode and cathode, respectively. On the anode side the OER follows an alkaline pathway, while on the cathode the HER follows an acidic pathway. Compared to the conventional BPMWE, where an AEM laminates with a PEM to form the bipolar junction,^20^ the BPIWE minimizes the water transport resistance by allowing water feed only to the anode, therefore producing dry hydrogen on the cathode. BPIWEs can also possibly create more bipolar interfaces due to a more tortuous anode catalyst layer compared to the planar bipolar interface in BPMWEs.

**Figure 1:**
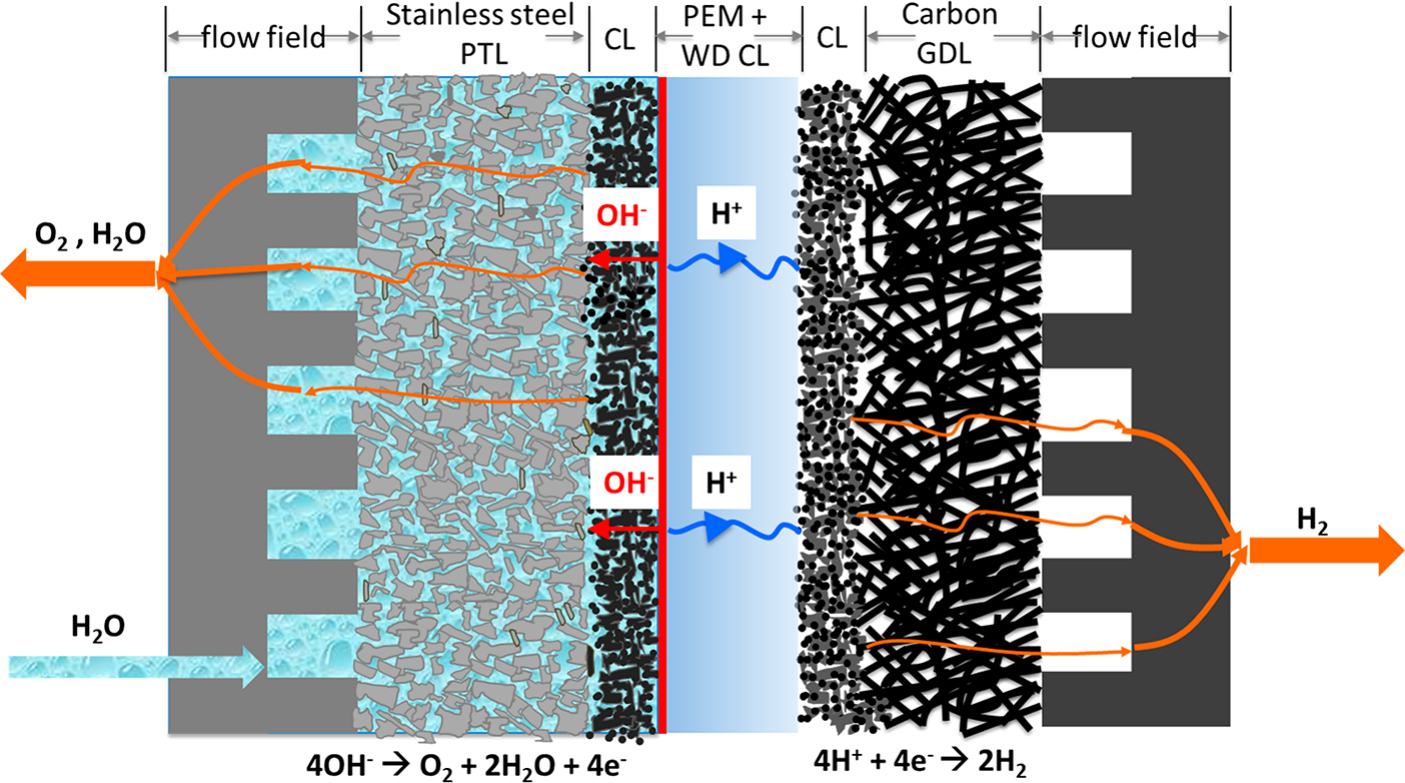
Schematic depiction of a bipolar-interface water electrolyzer. The red zone next to the PEM defines the WD catalyst layer.

One of the first concerns for the concept of a BPIWE is whether there exists an actual bipolar interface, namely, whether the anode’s OER follows an alkaline or acidic pathway. A similar BPIWE recently reported by Thiele and co-workers^21^ used IrO_*x*_ as both the anode OER and WD catalysts and demonstrated performance comparable to that of state-of-the-art PEMWEs.^22^ However, a WD catalyst layer made of IrO_*x*_ and Nafion could directly function as an anode for acidic OER, and the bipolar interface could be effectively circumvented. Besides, IrO_*x*_ has been shown to be an effective acidic OER catalyst even in the absence of proton-exchange ionomer (PEI) in PEMWEs,^23,24^ which could also contribute acidic OER though an anion-exchange ionomer (AEI) is used on the anode catalyst layer.

To verify the hypothesis of the existence of bipolar interfaces, we first judiciously chose non-precious-metal materials as both the anode OER catalyst and the WD catalyst. Here, Co_3_O_4_ is used as the anode OER catalyst, as it shows high electrolysis performance in pure-water-fed conditions, as demonstrated by Boettcher et al.^25^ As for the WD catalyst, there are many catalysts that have been tested to exhibit activity in BPMWEs.^18,26^ However, we think there are a few criteria that should be considered, particularly for BPIWEs: (i) As the WD reaction produces OH^–^ and H^+^, suggesting that both high-pH and low-pH domains can exist within the bipolar interfaces, the WD catalyst is required to be chemically stable in both high alkalinity and high acidity conditions. Therefore, transition-metal oxides or hydroxides such as NiO,^26^ Co_2_O_3_,^26^ and Al(OH)_3_^16^ could face durability concerns. (ii) Though WD catalysts are not directly involved in the OER, the potential at the bipolar interfaces can still be elevated,^19^ where corrosion can occur for carbon-based materials, such as graphene oxide.^17^ We therefore use TiO_2_, a common catalyst from previous literature,^19^ as the WD catalyst at the bipolar interfaces.

The Co_3_O_4_ and TiO_2_ catalyst materials were characterized by X-ray diffraction (XRD, Figure 2a). The diffraction peaks at 18.91°, 31.23°, 36.91°, 38.62°, 44.96°, 59.4°, and 65.29° can be assigned to the (111), (220), (311), (222), (400), (511), and (440) facets for Co_3_O_4_.^27,28^ The TiO_2_ XRD confirms the dominant anatase phase (JCPDS card no. 21-1272) with minor rutile phase (JCPDS card no. 21-1276) of P25 TiO_2_.^29^ Transmission electron microscopy (TEM) shows that Co_3_O_4_ has a particle size of around 20 nm with a 2 nm surface amorphous layer. The Co_3_O_4_ is fabricated into a porous transport electrode (PTE) via coating the catalyst ink (Co_3_O_4_ + AEI) on stainless steel PTLs (Figure 2f). The TiO_2_ is spray-coated on one side of the PEM and Pt/C is spray-coated on the other side as the cathode catalyst layer, as indicated by the scanning electron microscopy (SEM) images (Figure 2g).

**Figure 2:**
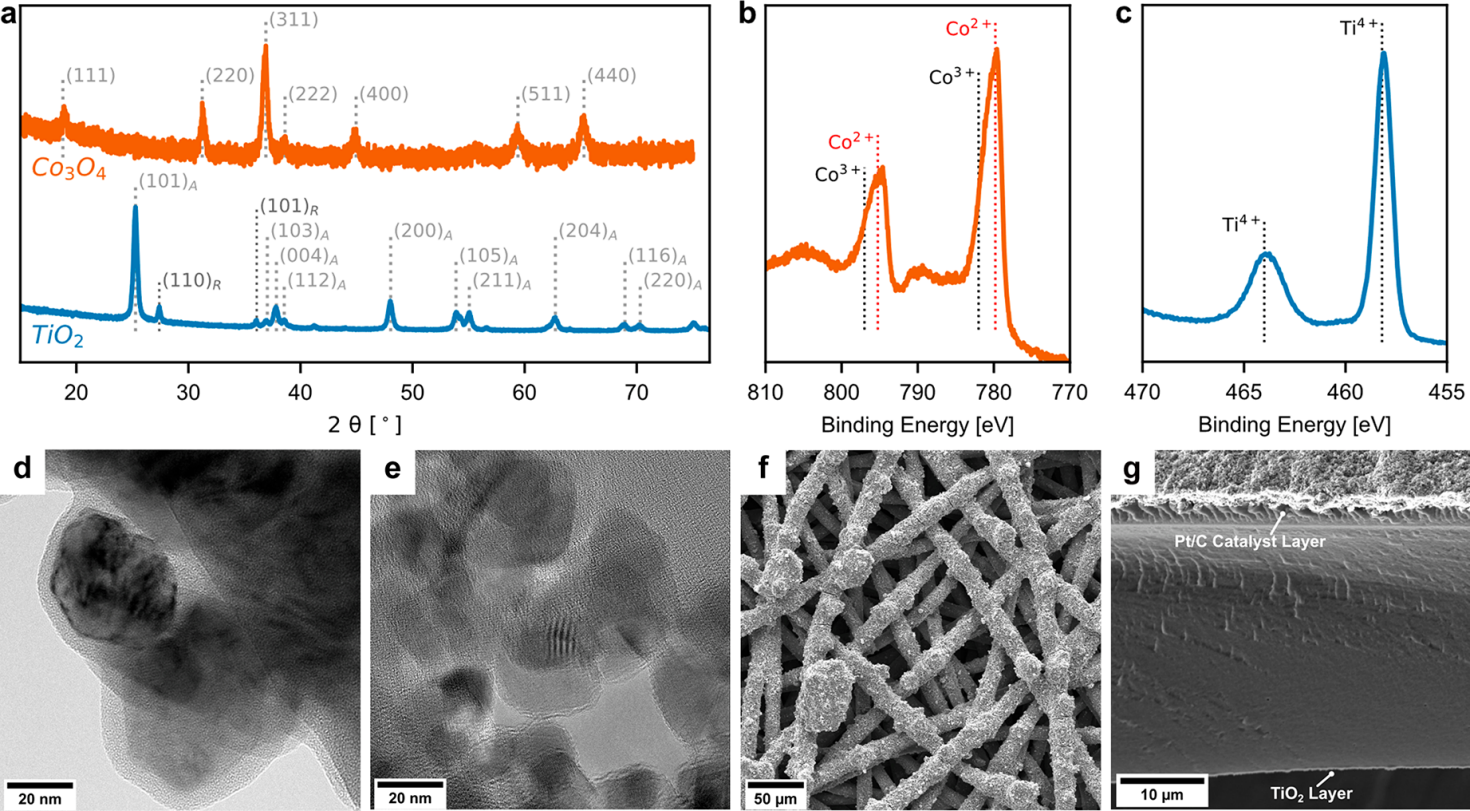
(a) X-ray diffraction (XRD) patterns of cobalt oxide (Co_3_O_4_) and titanium dioxide (TiO_2_). Subscripts on the TiO_2_ XRD peak assignments signify the anatase (A) or rutile (R) phase. X-ray photoelectron spectra of (b) Co_3_O_4_ and (c) TiO_2_. Transmission electron microscopy (TEM) images of (d) Co_3_O_4_ particles and (e) TiO_2_ particles. (f) Top-view scanning electron microscopy (SEM) image of the Co_3_O_4_ anode porous transport electrode. (g) Cross-section SEM image of half catalyst-coated membrane with a Pt/C cathode catalyst layer (top) and TiO_2_ water dissociation catalyst layer (bottom).

We then assembled the Co_3_O_4_ PTE and half catalyst-coated membrane (CCM) into a BPIWE to test its water-electrolysis performance. As shown in Figure 3, the BPIWE with non-precious-metal materials as OER and WD catalysts exhibits excellent performance with pure water fed to the anode, realizing one of the best-reported performances in zero-gap bipolar-based water electrolyzers (Table S1).^16−20,26^ We also repeated the measurement in three independent experiments to ensure reproducibility (Figure S1). To further verify the existence of a bipolar interface, we assembled a PEMWE by replacing the anode AEI (Piperion A) with PEI (Nafion) while keeping all other cell components and testing conditions unchanged. If the anode of BPIWE follows the acidic OER rather than alkaline OER pathway, one would expect the PEMWE to perform significantly better than the BPIWE, while the experimental result shows the exact opposite (Figure 3). These results could suggest that the anode follows a dominant alkaline OER pathway rather than an acidic pathway, which in turn indicates the formation of bipolar interfaces between the alkaline anode and PEM in the proposed BPIWE.

**Figure 3:**
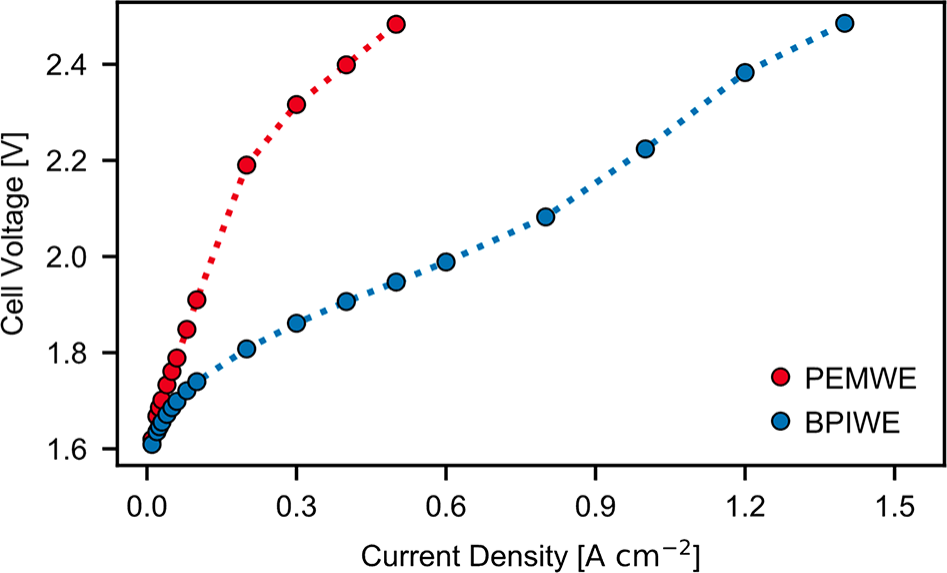
Water-electrolysis performance comparison of Co_3_O_4_ in BPIWE (blue curve) and PEMWE (red curve). Testing conditions: DI water fed to anode at 80 °C; anode, 1.3 ± 0.1 mg_Co_3_O_4__ cm^–2^; cathode, 0.1 mg_Pt_ cm^–2^ (Pt/C); WD loading, 6 μg cm^–2^.

To further demonstrate the superiority of the BPIWE, we compare it to the conventional BPMWE, where a PEM (Nafion 212, 50 μm) laminates with an AEM (Versogen, 40 μm) with TiO_2_ as WD catalyst at a loading of 6 μg cm^–2^. As shown in Figure 4a, the BPIWE shows enhanced water-electrolysis performance compared to the BPMWE. The BPMWE shows significantly higher high-frequency resistance (HFR) compared to the BPIWE (Figure 4b), probably due to the higher thickness of the AEM. Electrochemical impedance spectroscopy (EIS) of the BPMWE is generally accepted to exhibit two semicircles, one of which is associated with the WD reaction, and the other is associated with the anodic reaction.^19,30^ It is worth noting that the BPIWE shows only one semicircle compared to the two semicircles shown for the BPMWE (Figure 4b). The disappearance of the semicircle associated with the WD reaction indicates that the WD reaction is “fast” enough compared to the OER at the current where EIS is measured, which could suggest an expedited WD reaction in the BPIWE compared to the BPMWE. This is likely due to an enhanced electric field contributed by the bipolar interface between the anode and PEM where most of the voltage drop occurs, although more detailed studies are needed to truly deconvolute the effects.

**Figure 4:**
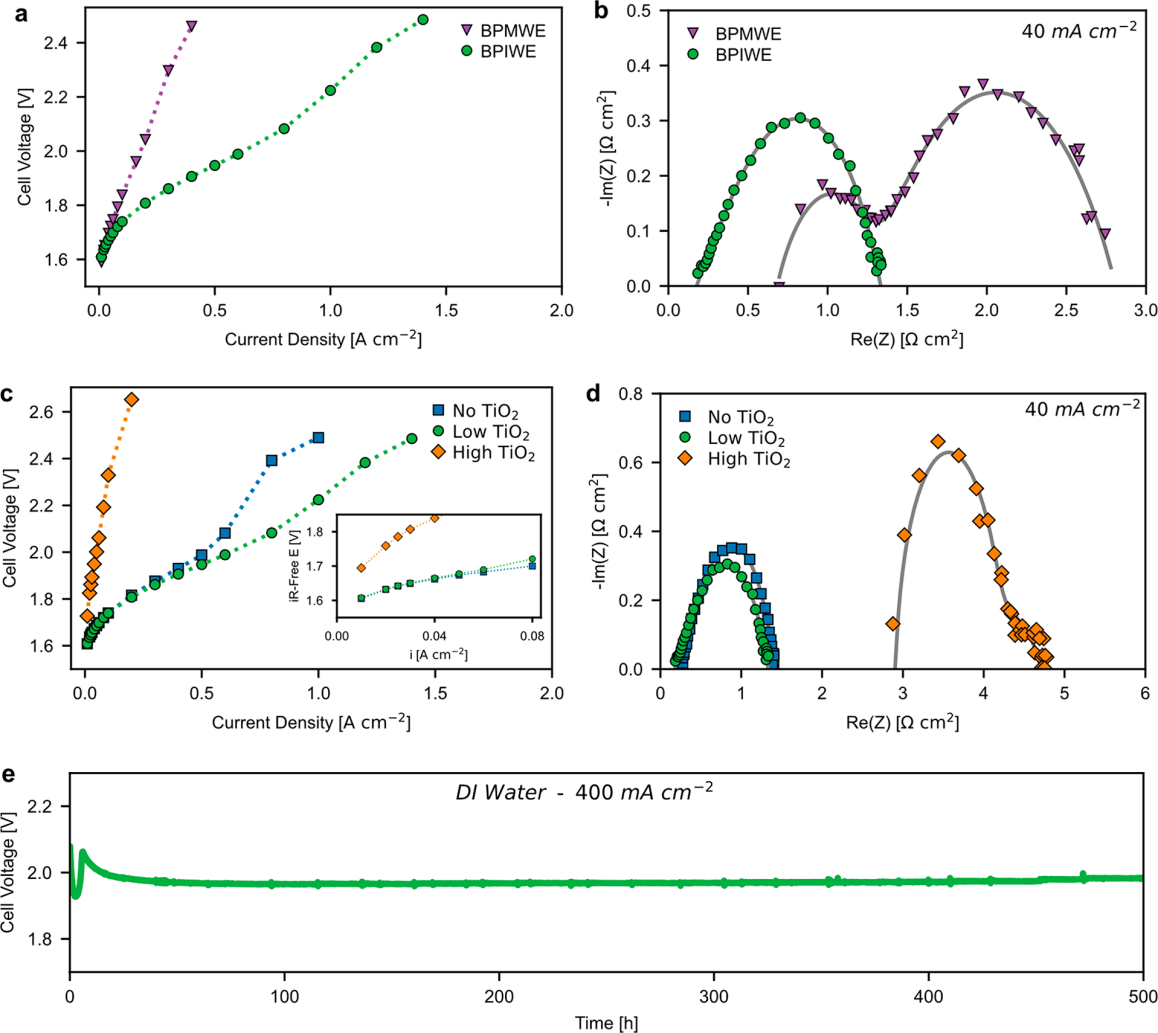
(a) Polarization curves and (b) electrochemical impedance spectroscopy (EIS) comparison between BPIWE (●) and BPMWE (▼). (c) Polarization curves and (d) EIS comparison of BPIWEs among three TiO_2_ WD catalysts loadings: no TiO_2_ WD catalyst (■), low TiO_2_ loading (6 μg cm^–2^) (●), and high TiO_2_ loading (60 μg cm^–2^) (◆). Inset shows the kinetic region. (e) Chronopotentiometry curve of the BPIWE at a current density of 400 mA cm^–2^ with DI water fed only to the anode at 80 °C. Initial voltage fluctuation was due to loss of water bath temperature. Anode: 1.3 ± 0.1 mg_Co_3_O_4__ cm^–2^. Cathode: 0.1 mg_Pt_ cm^–2^ (Pt/C).

To investigate how the TiO_2_ WD catalyst can impact the BPIWE’s performance, the TiO_2_ loadings were varied in three typical ranges (Figure 4c): no-TiO_2_ (0 mg cm^–2^), low-TiO_2_ (6 μg cm^–2^), and high-TiO_2_ (60 μg cm^–2^). The comparison between low-TiO_2_ and no-TiO_2_ indicates a voltage-dependent behavior of the WD reaction catalyzed by TiO_2_: at low current densities (0–80 mA cm^–2^) or low cell voltage, there is no obvious difference between the low-TiO_2_ and no-TiO_2_ (inset of Figure 4c), indicating that the electric field can sustain water self-dissociation and the TiO_2_ WD catalyst does not play a significant role in catalyzing WD, while at higher current densities or higher cell voltage, the BPIWE with WD catalyst demonstrates better performance, indicating that the WD rate catalyzed by TiO_2_ starts to play an important role. This interesting phenomenon is likely due to the water reorganization energy difference at different electric fields,^31^ which may manifest itself as a voltage-dependent activity of TiO_2_ for WD reactions. Besides, a higher electric field can also shift the equilibrium for WD (H_2_O ↔ OH^–^ + H^+^) and suppress the backward reaction via the second Wein effect, which potentially helps enhance the WD activity of TiO_2_.^32^ As TiO_2_ loading increases, there is a performance penalty due to a significant increase in the HFR from EIS measurements (Figure 4d). This is likely due to the fact that a high TiO_2_ loading increases the WD catalyst-layer thickness (2300 nm vs 250 nm) (Figures S2 and S3), which essentially forms a physical barrier to conduct charges (H^+^ and OH^–^) after the WD reaction. The EIS data also shows that the ionic resistance induced by the WD catalyst layer will be reflected in the HFR, which is contrary to what has been seen in BPMWEs.^19^ Besides, higher WD catalyst loading increases the thickness of the bipolar junction, which reduces the localization and thus the strength of the electric field, lessening the beneficial impact on the WD reactions, as also inferred from the distortion of the EIS (Figure 4d). The reduced electric field impacts electrode kinetics even at low current (inset of Figure 4c). These results suggest that a proper balance among WD catalytic effect, electric field, and ionic conductivity needs to be maintained at the bipolar interface for BPIWEs.

A longevity test was conducted by holding the cell current at 400 mA cm^–2^, with pure water recirculating only on the anode side at a rate of 25 mL min^–1^. Matching the high beginning-of-life performance as discussed above, the BPIWE also exhibited negligible performance degradation up to 500 h (Figure 4e). To our knowledge, this is the best-reported pure-water-fed bipolar water electrolyzer durability in MEA configurations (Table S2).^16−18,20,26^ The small voltage spikes were due to replenishing water in the water bath, which led to temporary temperature fluctuations. Excellent durability of another BPIWE at higher current densities (500 mA cm^–2^) was further demonstrated using an anode at higher catalyst loadings (2.0 ± 0.1 mg_Co_3_O_4__ cm^–2^) for 120 h (Figure S4), which also exhibited lower cell voltage compared to Figure 4e.

In summary, we demonstrate the feasibility of achieving high-performing and durable BPIWEs by eliminating the AEM in the conventional BMPWEs, which generates a high electric field and reduces ohmic resistance. The performance difference between PEMWEs and BPIWEs using the same Co_3_O_4_ material as anode OER catalyst confirms the existence of a bipolar interface between the alkaline anode and the PEM. The electric field can sustain water self-disassociation to a certain rate, whereas a WD catalyst (TiO_2_) is necessary to support BPIWE operation at high current densities. The results also demonstrate that a balance needs to be maintained among the WD reaction rate, electric field, and ionic conductivity when determining WD catalyst loadings. Overall, this work provides an efficient and novel approach to achieve high-performing water electrolyzers and excellent longevity at intermediate current density and dry-cathode conditions without supporting electrolytes using earth-abundant anode materials.

## Supporting Information Available

Experimental details, electrochemical measurements and materials characterizations, performance and durability comparison among different bipolar water electrolyzers, and SEM images (PDF)
