# Supplementary material for: Engineering Bipolar Interfaces for Water Electrolysis Using Earth-Abundant Anodes
Source: ACS Energy Lett. 2023 Nov 30;8(12):5275–80. doi: 10.1021/acsenergylett.3c02351 (PMC10714389; doi:10.1021/acsenergylett.3c02351)
Supplement: Supplementary file 1 — nz3c02351_si_001.pdf [file nz3c02351_si_001.pdf]

Supporting Information

Engineering bipolar interfaces for water electrolysis using earth-abundant anodes

Andrew W. Tricker[1, #], Jason K. Lee[1, #], Finn Babbe[1], Jason R. Shin,[1,2] Adam Z. Weber[1], Xiong Peng[1*]

[1]Energy Storage and Distributed Resources Division, Lawrence Berkeley National Laboratory, Berkeley, CA 94720, USA

[2]Department of Chemical & Biomolecular Engineering, University of California Berkeley, Berkeley, CA 94720, USA

[#]These authors contributed equally

AUTHOR INFORMATION

Corresponding Author

*Xiong Peng: xiongp@lbl.gov

Experimental Section

Chemicals and Materials

Cobalt oxide nanoparticles (Co_3O_4, >99.5%, 30-50 nm) was purchased from U.S. Research Nanomaterials, Inc (Houston, TX). Titanium dioxide nanoparticles (Aeroxide® P25, ≥99.5%, 21 nm) were purchased from Sigma Aldrich. Platinum supported on carbon (10V50E, 46.8 wt%) was purchased from Tanaka Kikinzoku Kogyo (TKK, Chiyoda-ku, Tokyo). PiperION A ionomer dispersion (5 wt%) and membrane (30 μm) was purchased from Versogen (Delaware). Nafion 212 membrane and D521 ionomer dispersion (5 wt%) was purchased from Ion Power (Delaware). Toray-120 (5 wt% PTFE) carbon paper was purchased from Fuel Cell Store (Texas). Stainless-steel fiber PTLs (10FP3) were purchased from Bekaert (Belgium). DI water (18.2 MΩ) was generated in-house from a Milli-Q system (Millipore).

Materials characterization

The surface morphology of the PTEs and MEAs were characterized using scanning electron microscopy (FEI Quanta FEG 250). Freshly prepared samples were placed into the specimen chamber under high vacuum conditions ($< 2 \times 10^{-5}$ Torr). The energy level of the beam was set to 10 kV. To obtain cross sectional images, samples were fractured under liquid nitrogen, and were carefully mounted onto the substrate. The X-ray diffraction (XRD) of the cobalt oxide and titanium dioxide was analyzed using a Rigaku Smartlab X-ray diffractometer equipped with a HyPix-3000 high energy resolution 2D multidimensional semiconductor detector. The powder samples were placed on the sample holder. The XRD measurements were performed by setting the same Rigaku

SmartLab diffractometer to Bragg-Brentano mode at room temperature. The surface compositions of cobalt oxide and titanium dioxide were characterized using X-ray photoelectron spectroscopy (XPS) Kratos Axis Ultra DLD system at takeoff angles of 0° and 60° relative to the surface normal. The measurements were conducted under room temperature and ultrahigh vacuum of 7.5×10^{-9} Torr. A monochromatic Al Kα source (hν = 1486.6 eV) was used to excite the core level electrons of the material. Spectral analysis was performed using CasaXPS analysis software. Transmission electron microscopy (TEM) imaging was performed on a JEOL-2100 electron microscope (JOEL Ltd.). Images were collected with an accelerating voltage of 200 kV and a beam current of ~16 pA.

Anode porous-transport-electrode fabrication

For anode inks, 100 mg of the cobalt oxide catalyst was added to 10 mL of a 1:9 v/v water/nPA mixture and sonicated for 10 min using a horn sonicator (CEX500, Cole-Parmer) at a power of 38%. Afterwards, 600 mg of 5 wt% PiperION A dispersion (I:Cat = 0.20) was added to the ink, and bath sonicated in an ice bath for at least 30 min. The porous transport electrodes were fabricated using a handheld airbrush (Iwata). The bare PTLs were cut to a precise size with an area of 30.25 cm^2 and massed before spraying. The inks were loaded into a hand-held airbrush connected to house nitrogen and deposited on the substrate over many passes until the desired loading was reached. Figure S5 shows SEM images of the PTE catalyst layer.

Fabrication of cathode catalyst layer and water dissociation catalyst layer

For the fabrication of cathode catalyst layer and water dissociation catalyst layer, ultrasonic spray coating method was used. The cathode catalyst ink was deposited onto the Nafion 212 membrane to fabricate a half-CCM, and water dissociation catalyst ink was deposited onto the other side of the Nafion 212 membrane to fabricate the water dissociation catalyst layer. The ink for the cathode side was prepared by mixing platinum supported by carbon (TEC10V50E 46.8% Pt, Tanaka) with DI water and n-propanol at a volumetric ratio of 1:1 and adding PFSA ionomer solution (Nafion 5 wt%, Ion Power D521) until ionomer to catalyst ratio of 0.45 was achieved. The ink mixture was bath sonicated for 30 min at 10°C. Immediately after cathode ink was prepared, the Sono-Tek spray coater at 120 kHz sonication rate was used to deposit cathode catalyst onto the membrane over the vacuum plate at 80°C. The platinum loading on the cathode side was measured to be 0.1 mg_{Pt}/cm^2) using X-ray fluorescence.

Water dissociation catalyst inks were prepared by mixing commercially titanium dioxide catalysts with Milli-Q DI water, ethanol, and n-propanol at a volumetric ratio of 1:1:2. The catalyst ink was sonicated using a horn sonicator (CEX500, Cole-Parmer) at a power of 38% for 30 min under an ice bath. The Sono-Tek ultrasonic spray coater was used for deposition of the water dissociation catalyst ink with sonicating nozzle set to 120 kHz. The mass of titanium dioxide powders was reduced by a factor of 10 to fabricate the low-loaded water dissociation catalyst layer with other ink composition unchanged.

Electrolyzer-cell assembly and testing

A single electrolyzer-cell hardware (Fuel Cell Technology, FCT) with a platinum-coated titanium single parallel-channel flow field on the anode, and a single serpentine-channel graphite flow field on the cathode was used for all the experiments. On the cathode side, carbon paper without micro-porous layer (Toray 120) with 5% PTFE was used as the gas diffusion layer. 20% compression of

the cathode GDL was achieved by controlling the ethylene tetrafluoroethylene gaskets while anode gasket thickness was matched to the PTE thickness. Electrolyzer cells were torqued uniformly up to 4.5 Nm. The active areas were designed to be 5 cm^2 for all tests.

A potentiostat (VSP 300, Biologic) equipped with a 20 A booster was used for electrochemical analyses. In-house modified FCT test station was used for conducting electrolyzer testing. Milli-Q DI water was fed into the anode at 80˚C while cathode inlet was capped and produced hydrogen was vented through cathode outlet. The anode water was recirculated for the duration of the experiment. A rod heater was used to maintain the electrolyzer cell at 80°C. Polarization curve was measured at various currents with 20 s holds each current until cell voltage reached 2.5 V followed by measurement of galvanostatic EIS at each current step measured from polarization curves between 1 MHz and 100 mHz. The amplitude of the alternating current was optimized for each step to ensure a sufficient signal to noise ratio while maintaining a linear system response.

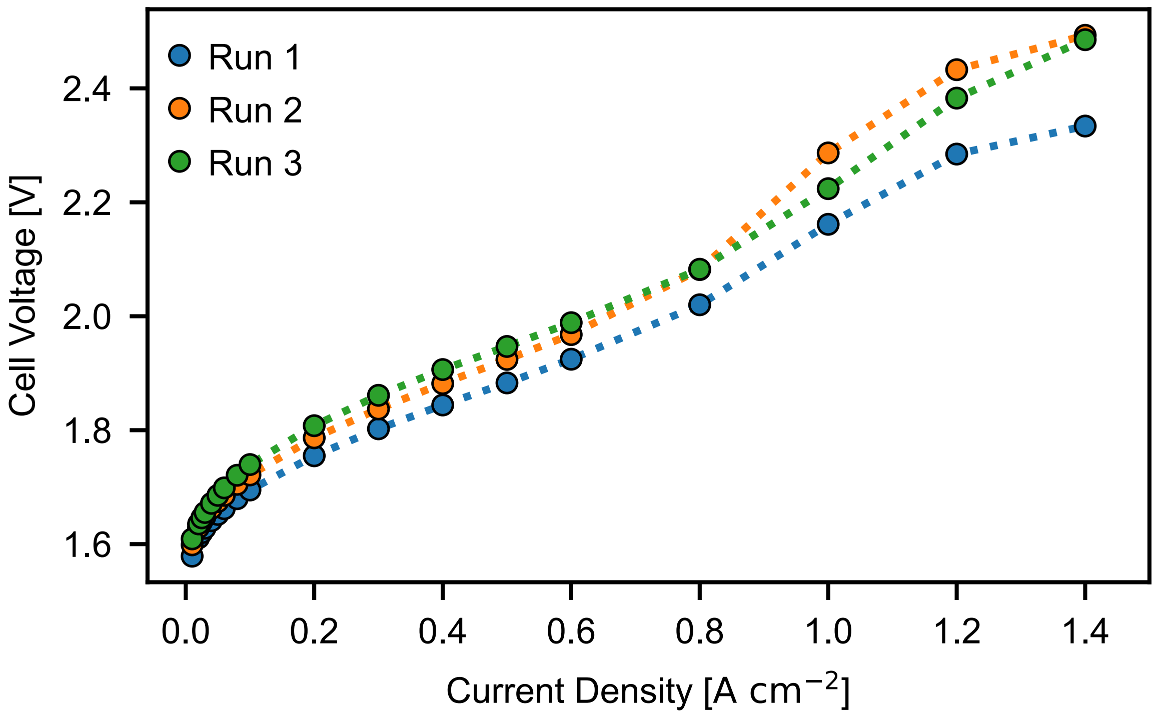

Figure S1. Reproducibility of the bipolar interface water electrolyzer (BPIWE) performance from three independent experiments. Experiential conditions for Run 1 and Run 2: Anode: 1.3±0.1 mg_{Co3O4} cm^{-2}, Cathode: 0.1 mg_{Pt} cm^{-2} (Pt/C). Proton exchange membrane: Nafion 212 and water dissociation catalyst: TiO_2 (6 µg cm^{-2}). Run 3 was conducted with an anode at 2.0±0.1 mg_{Co3O4} cm^{-2} with other conditions the same as Run 1 and Run 2. All tests were done with DI water fed only to anode side at 80 °C using 5 cm^2 single cell device.

Table S1. Summary of BPM/BPI water electrolyzer performance comparison.

Configuration	Electrocatalysts Anode/cathode	WDC	BPM/BPI	Temp [°C]	Anolyte	Catholyte	Current density [A cm^{-2}]	Voltage [V]	Ref
H-cell	n/a	Graphene Oxide	Cross-linked AEP | Nafion	RT	0.5 M KOH	0.5 M HCl	0.2	1.9*	[1]
H-cell	n/a	n/a	3D Junction BPM	RT	0.5 M KOH	0.5 M HCl	0.2	1.9*	[1]
H-cell	n/a	$Al(OH)_3$	3D Junction BPM (QPPO | SPEEK)	RT	0.5 M Na_2SO_4	0.5 M Na_2SO_4	1	0.9*	[2]
H-cell	n/a	Goethite $Fe^{+3}O(OH)$	QPPO | Nafion 212	RT	0.5 M NaCl	0.5 M NaCl	0.1	1.1*	[3]
MEA	IrO_2 | Pt	IrO_2 | NiO	Sustainion X37-50 | Nafion 212	50	DI Water	n/a	0.75	2.5	[4]
MEA	IrO_2 | Pt	TiO_2 | NiO	Sustainion X37-50 | Nafion 212	50	DI Water	n/a	1	3	[5]
MEA	IrO_2 | Pt	TiO_2 | NiO	Sustainion X37-50 | Nafion (3 μm)	50	DI Water	n/a	1	2.5	[5]
MEA	IrO_2 | Pt	TiO_2 P25	PiperION A-40 | Nafion 212	55	DI Water	n/a	0.5	2	[6]
MEA	Co_3O_4 | Pt/C	TiO_2 P25	PiperION A (AEI) | Nafion 212	80	DI Water	n/a	1.5	2.5	This Work

*Transmembrane Voltage, WDC = water dissociation catalyst

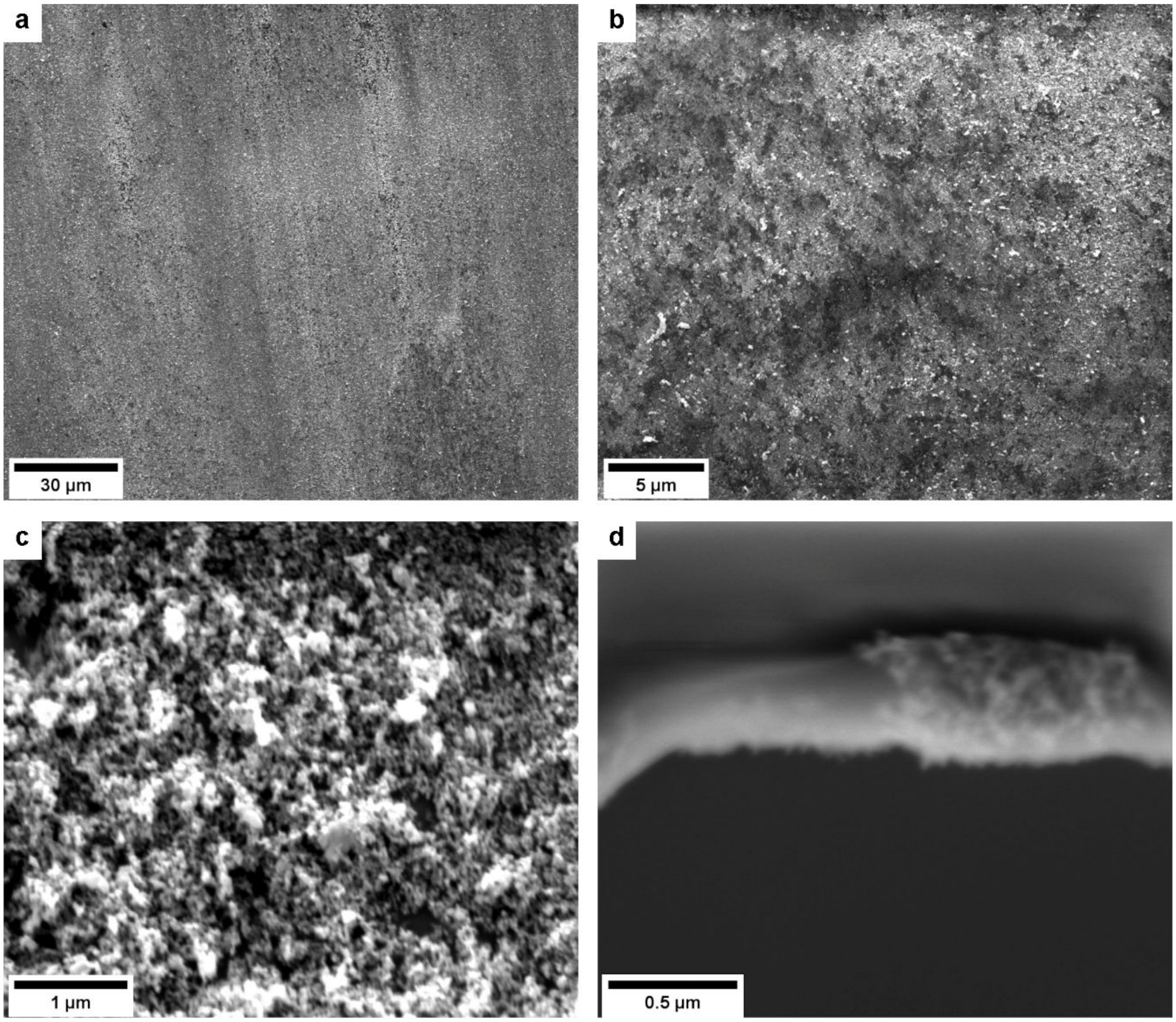

Figure S2. SEM images of the low-loading TiO_2 WD catalyst layer (6 µg cm^{-2}): a-c) top-down images at varying magnification, d) cross-section image.

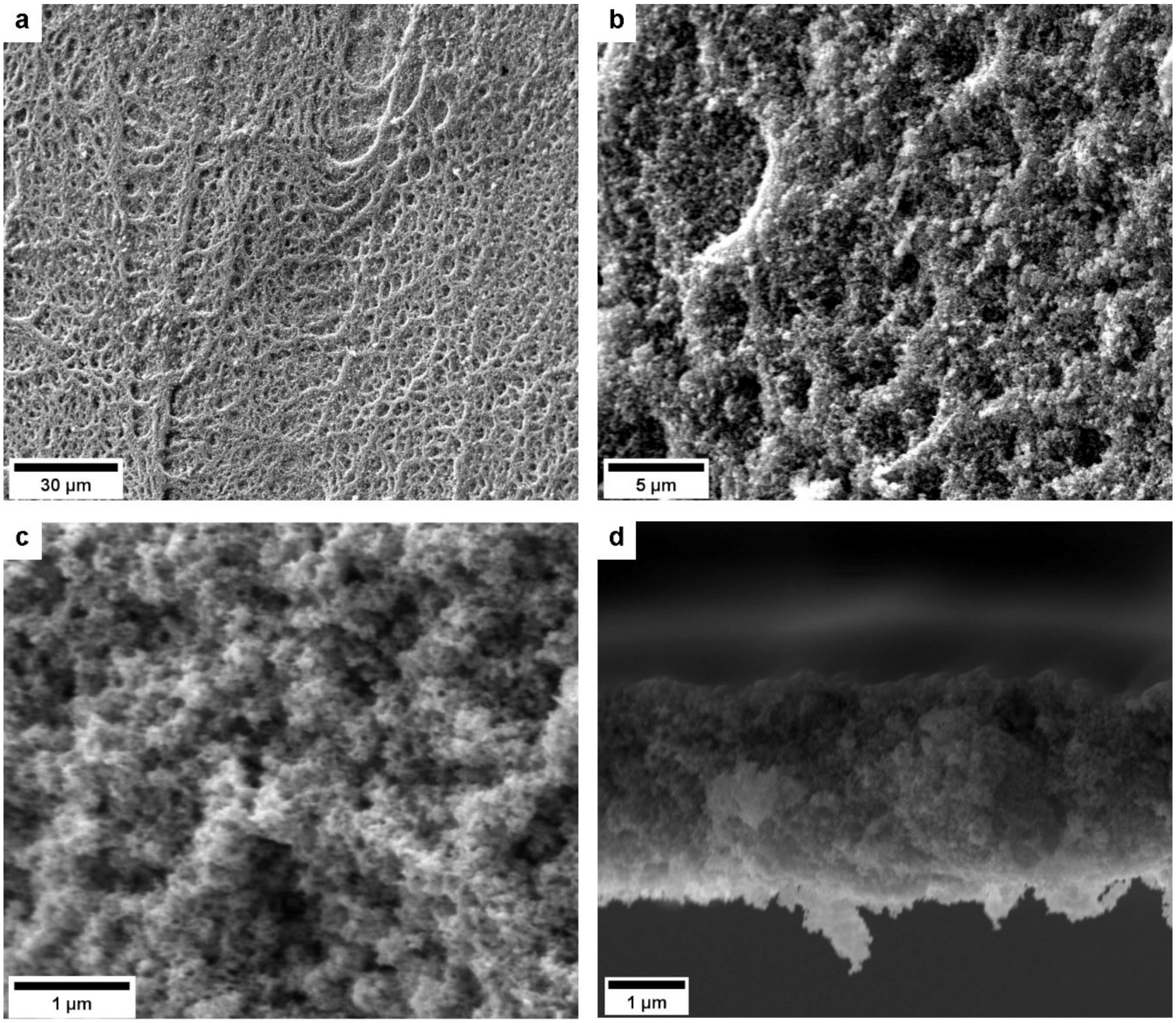

Figure S3. SEM images of the high-loading TiO_2 WD catalyst layer (60 µg cm^{-2}): a-c) top-down images at varying magnification, d) cross-section image.

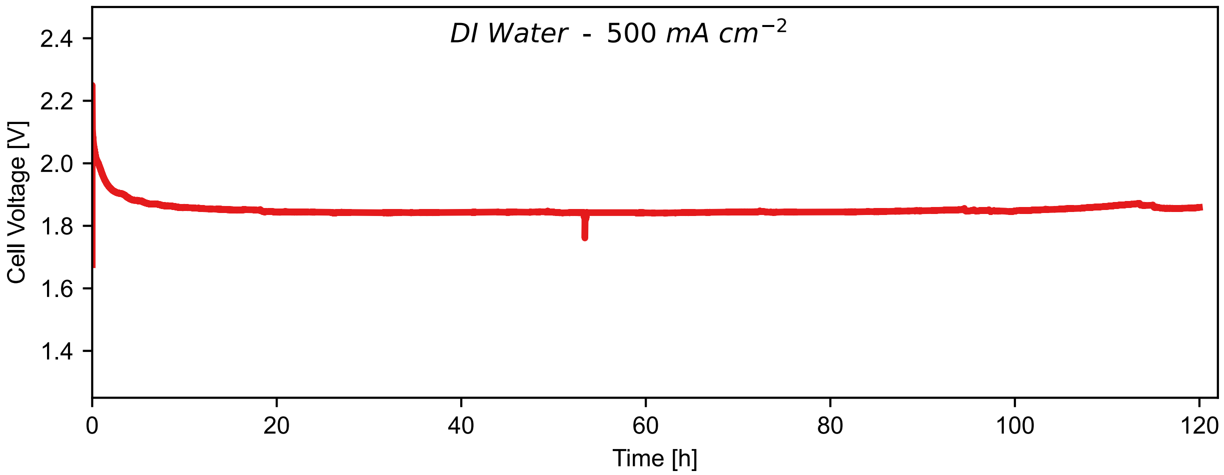

Figure S4. Durability of the BPIWE during current holds at 500 mA cm^{-2}. Anode: 2.0±0.1 mg_{Co3O4} cm^{-2}, Cathode: 0.1 mg_{Pt} cm^{-2} (Pt/C); Proton exchange membrane: Nafion 212 and water dissociation catalyst: TiO_2 (6 µg cm^{-2}). DI water was fed to anode at 80 °C using 5 cm^2 single cell device. This was the same membrane electrode assembly as Run 3 in Figure S1.

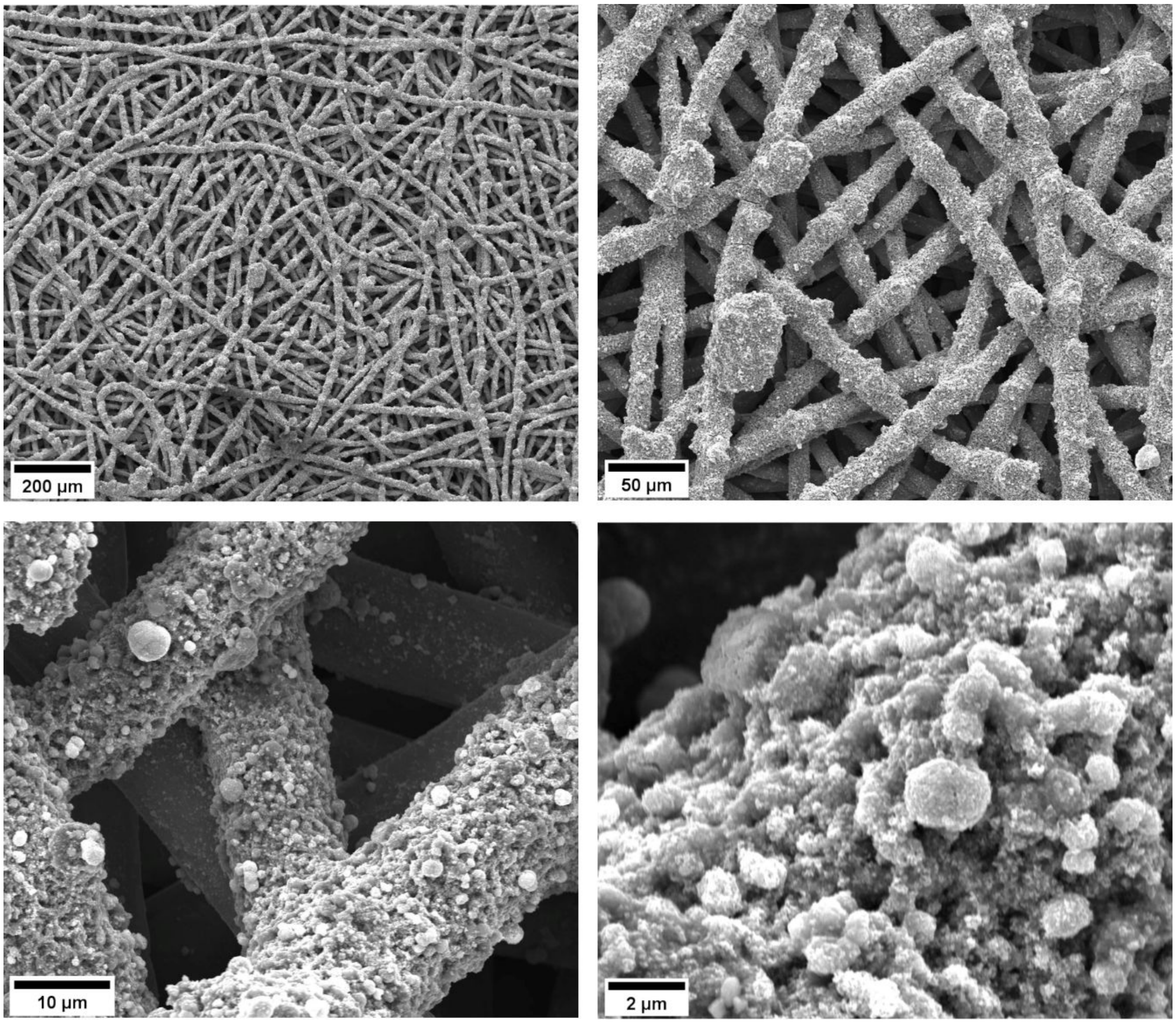

Figure S5. Scanning electron microscopy (SEM) images of the cobalt oxide porous transport electrode (PTE) catalyst layer.

Table S2. Summary of BPM/BPI water electrolyzer durability comparison.

Configuration	Electrocatalysts Anode/cathode	WDC	BPM/BPI	Temp [°C]	Anolyte	Catholyte	Duration [h]	Current Density [A cm^{-2}]	Degradation Rate [µV h^{-1}]	Ref
H-cell	n/a	Graphene Oxide	Cross-linked AEP | Nafion	RT	0.5 M KOH	0.5 M HCl	10	0.1	n.r.*	[1]
H-cell	n/a	nGO	3D Junction BPM (QPPO | SPEEK)	30	0.5 M Na_2SO_4	0.5 M Na_2SO_4	60	0.8	Negligible*	[2]
H-cell	n/a	Goethite $Fe^{+3}O(OH)$	QPPO | Nafion 212	RT	0.5 M NaCl	0.5 M NaCl	140	0.1	3*	[3]
MEA	IrO_2 | Pt	IrO_2 | NiO	Sustainion X37-50 | Nafion 212	50	DI Water	n/a	4	0.5	~10,000	[4]
MEA	IrO_2 | Pt	TiO_2 | NiO	Sustainion X37-50 | Nafion (3 µm)	50	DI Water	n/a	4	1	~20,000	[5]
MEA	Co_3O_4 | Pt/C	TiO_2 P25	PiperION (AEI) | Nafion 212	80	DI Water	n/a	500	0.4	30	This Work
MEA	Co_3O_4 | Pt/C	TiO_2 P25	PiperION (AEI) | Nafion 212	80	DI Water	n/a	120	0.5	350	This Work

*Transmembrane Voltage, n.r. = not reported, WDC = waster dissociation catalyst

References

(1) Yan, Z.; Zhu, L.; Li, Y. C.; Wycisk, R. J.; Pintauro, P. N.; Hickner, M. A.; Mallouk, T. E. The balance of electric field and interfacial catalysis in promoting water dissociation in bipolar membranes. *Energy Environ. Sci.* **2018**, *11* (8), 2235-2245.

(2) Powers, D.; Mondal, A. N.; Yang, Z.; Wycisk, R.; Kreidler, E.; Pintauro, P. N. Freestanding bipolar membranes with an electrospun junction for high current density water splitting. *ACS Appl. Mater. Interfaces* **2022**, *14* (31), 36092-36104.

(3) Shehzad, M. A.; Yasmin, A.; Ge, X.; Ge, Z.; Zhang, K.; Liang, X.; Zhang, J.; Li, G.; Xiao, X.; Jiang, B. Shielded goethite catalyst that enables fast water dissociation in bipolar membranes. *Nat. Commun.* **2021**, *12* (1), 1-13.

(4) Oener, S. Z.; Foster, M. J.; Boettcher, S. W. Accelerating water dissociation in bipolar membranes and for electrocatalysis. *Science* **2020**, *369* (6507), 1099-1103.

(5) Oener, S. Z.; Twight, L. P.; Lindquist, G. A.; Boettcher, S. W. Thin cation-exchange layers enable high-current-density bipolar membrane electrolyzers via improved water transport. *ACS Energy Lett.* **2020**, *6* (1), 1-8.

(6) Chen, L.; Xu, Q.; Oener, S. Z.; Fabrizio, K.; Boettcher, S. W. Design principles for water dissociation catalysts in high-performance bipolar membranes. *Nat. Commun.* **2022**, *13* (1), 1-10.
